# Comparing Artificial Intelligence Guided Image Assessment to Current Methods of Burn Assessment

**DOI:** 10.1093/jbcr/irae121

**Published:** 2024-06-26

**Authors:** Justin J Lee, Mahla Abdolahnejad, Alexander Morzycki, Tara Freeman, Hannah Chan, Collin Hong, Rakesh Joshi, Joshua N Wong

**Affiliations:** Division of Plastic and Reconstructive Surgery, Department of Surgery, University of Alberta, Edmonton, Alberta, T6G 2B7, Canada; Skinopathy Inc., Toronto, Ontario, M2N1N5, Canada; Division of Plastic and Reconstructive Surgery, Department of Surgery, University of Alberta, Edmonton, Alberta, T6G 2B7, Canada; Division of Plastic and Reconstructive Surgery, Department of Surgery, University of Alberta, Edmonton, Alberta, T6G 2B7, Canada; Skinopathy Inc., Toronto, Ontario, M2N1N5, Canada; Skinopathy Inc., Toronto, Ontario, M2N1N5, Canada; Skinopathy Inc., Toronto, Ontario, M2N1N5, Canada; Division of Plastic and Reconstructive Surgery, Department of Surgery, University of Alberta, Edmonton, Alberta, T6G 2B7, Canada

**Keywords:** burn assessment, laser Doppler imaging, artificial intelligence, convoluted neural network, Boundary Attention Mapping

## Abstract

Appropriate identification of burn depth and size is paramount. Despite the development of burn depth assessment aids [eg, laser Doppler imaging (LDI)], clinical assessment, which assesses partial-thickness burn depth with 67% accuracy, currently remains the most consistent standard of practice. We sought to develop an image-based artificial intelligence system that predicts burn severity and wound margins for use as a triaging tool in thermal injury management. Modified EfficientNet architecture trained by 1684 mobile-device-captured images of different burn depths was previously used to create a convoluted neural network (CNN). The CNN was modified to a novel boundary attention mapping (BAM) algorithm using elements of saliency mapping, which was used to recognize the boundaries of burns. For validation, 144 patient charts that included clinical assessment, burn location, total body surface area, and LDI assessment were retrieved for a retrospective study. The clinical images underwent CNN-BAM assessment and were directly compared with the LDI assessment. CNN using a 4-level burn severity classification achieved an accuracy of 85% (micro/macro-averaged receiver operating characteristic scores). The CNN-BAM system can successfully highlight burns from surrounding tissue with high confidence. CNN-BAM burn area segmentations attained a 91.6% accuracy, 78.2% sensitivity, and 93.4% specificity, when compared to LDI methodology. Results comparing the CNN-BAM outputs to clinical and LDI assessments have shown a high degree of correlation between the CNN-BAM burn severity predictions to those extrapolated from LDI healing potential (66% agreement).

CNN-BAM algorithm gives equivalent burn-depth detection accuracy as LDI with a more economical and accessible application when embedded in a mobile device.

## INTRODUCTION

Burn injuries are one of the leading causes of long-term morbidity worldwide associated with prolonged hospitalization, disfigurement, and disability. This is due to the complex pathophysiology of burn injuries, predisposing these patients to infection, hypothermia, hypovolemic shock, end-organ ischemia, and death. Advancements in the effective prevention, management, and treatment of burns carry significant implications for public health.

Burn severity is assessed based on the mechanism, total body surface area affected (TBSA%), and depth. This guides resuscitation and treatment decisions.^1^ Burn depth relates to healing potential and the projected risk of hypertrophic scar formation and functional contractures guides surgical decision-making.^1,2^ This is categorized into first-degree or superficial burns, involving only the epidermis; second-degree, which is subdivided into superficial partial thickness (SPT) and deep partial thickness (DPT) burns involving the dermis; third-degree or full-thickness burns, involving the epidermis and dermis; and fourth degree, which penetrates beyond the dermis into underlying tissues, including hypodermis, muscle fascia, and bone or other internal organs.^3^ As burn injury penetrates the dermis and beyond, the larger surface area injuries require clinical intervention to prevent acute and chronic complications such as shock, infection, hypertrophic scarring, and contractures.^2–4^ Therefore, accurate and timely assessment of burn severity is essential in burn management and significantly can impact patient prognosis.^1^

Unfortunately, clinical assessment of burn depth by even the most experienced burn surgeons has reported an accuracy of only 60%-75% for mixed-depth burns.^3,5,6^ Given the impact of accurate assessments on treatment decisions and clinical outcomes, various adjunct tools and technologies have been developed to assist clinicians.^7,8^ Modalities explored for assessing burn depth include laser Doppler imaging (LDI), thermography, ultrasonography, nuclear magnetic resonance, near-infrared spectroscopy, and confocal microscopy. However, not all are equally applied in clinical practice due to the availability of equipment or limitations of training and environment.^5^ LDI, the most accurate of adjunct devices, has a sensitivity of 91% and up to 96% accuracy in predicting a patient’s recovery from a burn within a certain timeframe.^5,6^ However, barriers to using LDI in clinical contexts exist, including the limited availability of LDI equipment to major burn trauma centers and the high costs associated with the purchase and maintenance of the machine.^6,9^ Moreover, LDI accuracy increases over time, with 97% accuracy only being achieved at 5 days. This can delay the time to intervention or require an individual to accept a less accurate prediction for earlier intervention.^5^ These limitations are exacerbated in remote areas and low-resources settings, inciting a need for alternative accurate assessment tools that are rapid, accessible, and affordable for clinical application.

Applications of artificial intelligence (AI) or machine learning (ML) have garnered significant interest in this area, demonstrating potential for accessible and rapid burn assessment and prediction of wound healing.^10–16^

In a previous study, we developed a convolutional neural network (CNN) model, which learned from 1684 clinical burn images categorized into 4 depths. The purpose of this study was to compare the accuracy of a new CNN model compared to LDI assessment. We hypothesized that a CNN model can provide equivalent accuracy to that of LDI in burn depth assessment.

## METHODS

### Study design

As a part of standard clinical practice, each patient assessed in the Acute Burn Clinic at a Northern Canadian regional burn catchment center has photographs taken of their thermal injuries. Identifiable patient features are avoided, if possible, in these photos and images are maintained as part of their health records on EPIC Connect Care Media Manager for clinical decision-making. Patients who undergo an LDI assessment will also have their report uploaded to their records.

After appropriate approval was received from the Human Research and Ethics Board at the Regional Research Information Services System Pro00114696, de-identified clinical photos and LDI reports of all patients who underwent LDI assessment at the Acute Burn Clinic between December 2021 and July 2023 were collected for the study.

### Data collection and exploratory data analysis

Charts were identified through the Acute Burn Clinic records logged in EPIC Connect Care electronic medical records. Demographic data extracted included age, date of injury, clinical assessment of burn depth, TBSA, location of injury, LDI determination, treatment type, follow-up duration, days to recovery, and complications. In addition, patient skin tone was categorized using the Fitzpatrick scale by 3 separate assessors. These characteristics underwent exploratory data analysis to understand patient characteristics.

### Artificial intelligence pipeline

**Figure 1.**
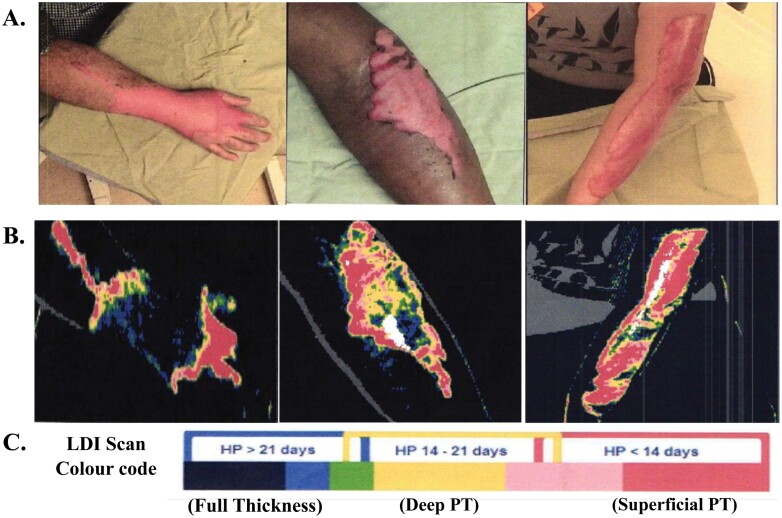
An Example of 2D Clinical Color Images and Complementary BloomLDI Scan Images from Patients. After Clinical Photo Is Obtained (A), LDI Scan Is Completed From the Same Angle and Distance From the Subject (B). LDI Scan Is Displayed in Color Code, Where Red Pixels Depict Healing Potential (HP) of Less Than 14 Days, Which Is Equivalent to Superficial Partial Thickness (PT) Injury; Yellow Pixels Depict HP of 14–21 Days, Which Is Equivalent to Deep PT; and Blue Pixels Depict HP Greater Than 21 Days, Which Is Equivalent to Full-Thickness Injury (C). Note: Green and Pink Pixels Depict the Thresholds Between FT/DPT and DPT/SPT, Respectively. Black Pixels Denote FT If on Skin of Patient

An AI pipeline was created which determines burn severity and identifies the region of the burn from 2D color images, as described by Olivier et al. (2022) and Abdolahnejad et al. (2023).^10,11^ Briefly, a CNN, using a pre-trained base architecture of EfficientNet-B7, was trained on de-identified 2D color images of skin burns, captured using a digital single lens reflex (DSLR) camera or mobile camera device. The CNN can classify 4 levels of burn severity and predicts the highest burn severity it recognizes within an image. By using select activation channels and information from neuronal layers from this CNN, a saliency mapping system, the boundary attention mapper (BAM), was created to identify the contours of a burn. To compare the accuracy of this BAM system to the non-invasive LDI method, LDI scans from patients were used to create a novel binary benchmark dataset, using the LDI scans and complementary 2D color images by conducting manual and semi-automated segmentations of the burn images (Figure 1).

### Comparative study and statistical analysis

The CNN performance for predicting burn severity was ascertained by performance metrics, such as F1 (harmonized accuracy), recall, and precision scores. An area under curve-receiver operating characteristic (ROC) analysis was also conducted for average micro/macro values. The statistical significance for determining regions of burns, between LDI and BAM methodologies, was done by pixel-wise comparisons of scans and BAM maps for 104 patients (184 LDI scans), and reported in terms of BAM accuracy, specificity, and sensitivity, with LDI accuracy considered to be at 100% value for all 3 metrics. Directly comparing clinical, LDI, and AI accuracy in predicting the severity of burns was not possible, as discussed later. However, a 3-way agreement inter-rater analysis for each patient (*n* = 138, using 241 LDI scans), for all 3 assessment methodologies was undertaken. Cohen’s kappa analysis was used to assess the inter-rater reliability of the assessment methods, using SPSS statistics software. Cohen’s kappa value demonstrates none to slight agreement at a kappa value of 0-0.20, fair agreement at 0.21-0.40, moderate agreement at 0.41-0.60, substantial agreement at 0.61-0.80, and excellent agreement at 0.81-1.00. Statistical power is determined at a *P* value less than .05.

## RESULTS

### Exploratory data analysis

#### Demographics

At our institution, initial burn assessments were completed through emergency department referrals or at the Acute Burn Clinic. Inclusion criteria included patients with undifferentiated burn depth injuries that received LDI assessment within 72-96 h. Exclusion criteria were patients with clear burn depth injuries or patients who did not have LDI images taken within the 72- to 96-h timeframe. Of all patients assessed between 2021 and 2023, 144 patients were selected. The age distribution of burn injury is demonstrated in Table 1.

**Table 1.** Demographics of Patients With Burn Injuries That Required LDI Assessment

Age range	Total
Child (0–9)	17
Adolescent (10–17)	9
18–30	30
31–50	48
51–70	32
>70	8
**Total**	**144**

#### Burn injury

Of 144 patients who underwent LDI assessment, burn size TBSA percentages ranged from < 1% to 70%. There were 92 patients with a TBSA of 1%-5%, 30 patients with a TBSA of 6%-10%, 12 patients with a TBSA of 11%-15%, 6 patients with a TBSA of 16%-20%, 2 patients with a TBSA of 21%-25%, and only 1 patient with a TBSA of 70%. No cases within our data presented with a TBSA between 26% and 69% or >70% that required LDI determination (Table 2).

**Table 2.** Total Body Surface Area (TBSA) and Location of Burn Injuries That Required LDI Assessment

TBSA percentage	Total number of patients
<1%	1
1–5%	92
6–10%	30
11–15%	12
16–20%	6
21–25%	2
70%	1
Total	144

The location of burn injury was highly variable among patients. There were 13 cases of burns to the face/head, 3 cases of burns to the neck, 16 cases of burns to the chest, 38 cases of burns to the arm, and 44 cases of burns to the hand. Other presentations included 8 burns to the abdomen, 9 to the back, 6 to the buttocks, and 3 to the groin. Finally, there were 28 presentations of burns to the leg and 26 burns involving the feet. The most common case for 21.2% of patients was multiple burn locations in 1 injury presentation. The hand was the most common single-site injury, occurring 15.9% of the time.

#### Fitzpatrick skin type

**Figure 2.**
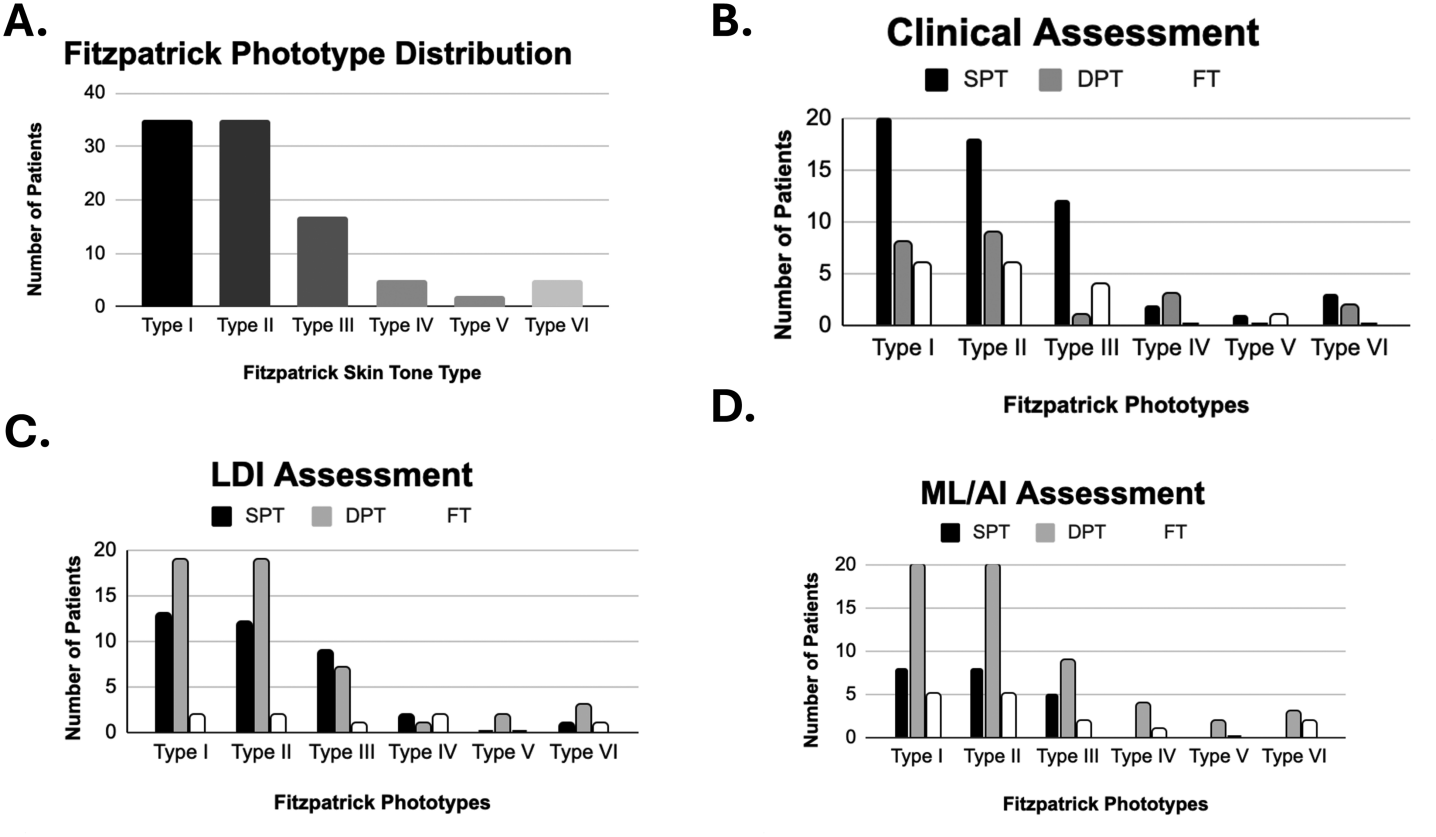
Fitzpatrick Scale Distribution of Patient Population with Thermal Injuries (A). Three Separate Assessors Determined the Fitzpatrick Type of Each Patient’s Skin Phototype. Type I Comprised 38% of 173 Images From 144 Patients, Type II at 32%, Type III at 18%, Type IV at 5.8%, Type V at 2.3%, and Type VI at 3.5%. Of Note, No Patient Had Tattoos in the Field of Burn Injury and/or LDI Assessment. Distribution of Burn Depth Assessments of Clinical, Laser Doppler Imaging (LDI), and Machine Learning/Artificial Intelligence (ML/AI) Assessments Based on Fitzpatrick Skin Type (B–D, respectively). Corresponding Values Are Displayed on Table 3

From 144 patients, there were 173 clinical images that were assessed as separate wound entities. This was due to a number of patients having multiple body areas injured at differing burn severity. Each clinical image was assessed by 3 independent assessors from the research team and given a Fitzpatrick scale. Conflicting categorizations were averaged and scored in the majority assessment. Type I comprised 66 of 173 images from 144 patients (38%), Type II at 56 (32%), Type III at 31 (18%), Type IV at 10 (5.8%), Type V at 4 (2.3%), and Type VI at 6 (3.5%). Of note, no patient had tattoos in the field of burn injury and/or LDI assessment (Figure 2A). Furthermore, the distribution of burn depth assessment using clinical, LDI, and AI based on the Fitzpatrick scale is displayed in Figure 2B–D and Table 3.

**Table 3.** Distribution of Burn Depth Assessments of Clinical, LDI, and AI Based on Fitzpatrick Scale

	Type I	Type II	Type III	Type IV	Type V	Type VI
Clinical assessments						
**SPT**	48	33	20	2	3	4
**DPT**	10	17	6	8	0	2
**FT**	8	6	5	0	1	0
**LDI assessments**						
**SPT**	21	24	17	4	0	1
**DPT**	40	27	12	2	3	4
**FT**	5	5	2	4	1	1
**AI assessments**						
**SPF**	1	0	1	0	0	0
**SPT**	14	14	9	1	0	0
**DPT**	39	36	18	8	3	4
**FT**	12	6	3	1	1	2

#### Assessment of burn depth

Almost all burn injuries initially undergo physician assessment. Of the 144 patients, several patients had burn injuries to multiple sites. Each individual site (eg, upper arm and chest) was captured as 2 separate LDI assessments. Exploratory data analysis revealed 2 LDI assessments initially received clinical assessment of superficial burn, 150 were assessed as superficial partial thickness, 65 were assessed as deep partial thickness, and 26 were assessed as full-thickness burn injuries (Table 4).

**Table 4.** Burn Depth Categorization of Burn Injuries From Clinical, LDI, and AI Assessment

Burn depth categorization	Clinical assessment	LDI assessments	AI assessments
SPT	150	69	42
DPT	65	136	156
FT	26	33	35

Three LDI scans could not be included in for AI analysis due to missing color images; 8 images were categorized as superficial burns by the AI.

LDI assessments of burn injuries are reported as healing estimates of < 14 days (equivalent to superficial partial thickness), 14-21 days (equivalent to deep partial thickness), and > 21 days (equivalent to full thickness). Our data showed 69 LDI assessments with < 14 days of healing potential, 136 LDI assessments with 14-21 days of healing potential, and 33 LDI assessments with > 21 days of healing potential (Table 4).

#### Artificial intelligence pipeline and statistical analysis

As described previously in Abdolahnejad et al. (2023), we validated the efficacy of the proposed pipeline by using 2 distinct datasets: a CNN training dataset and an LDI benchmark dataset. The CNN training dataset consisted of 1684 skin burn images, taken by mobile camera and DSLR devices, depicting 4 different levels of burn severity. The CNN trained on this first dataset exhibited notable performance, achieving an average F1 score of 78% and micro/macro-averaged ROC scores of 85% for the classification of the 4 burn severity levels.

**Figure 3.**
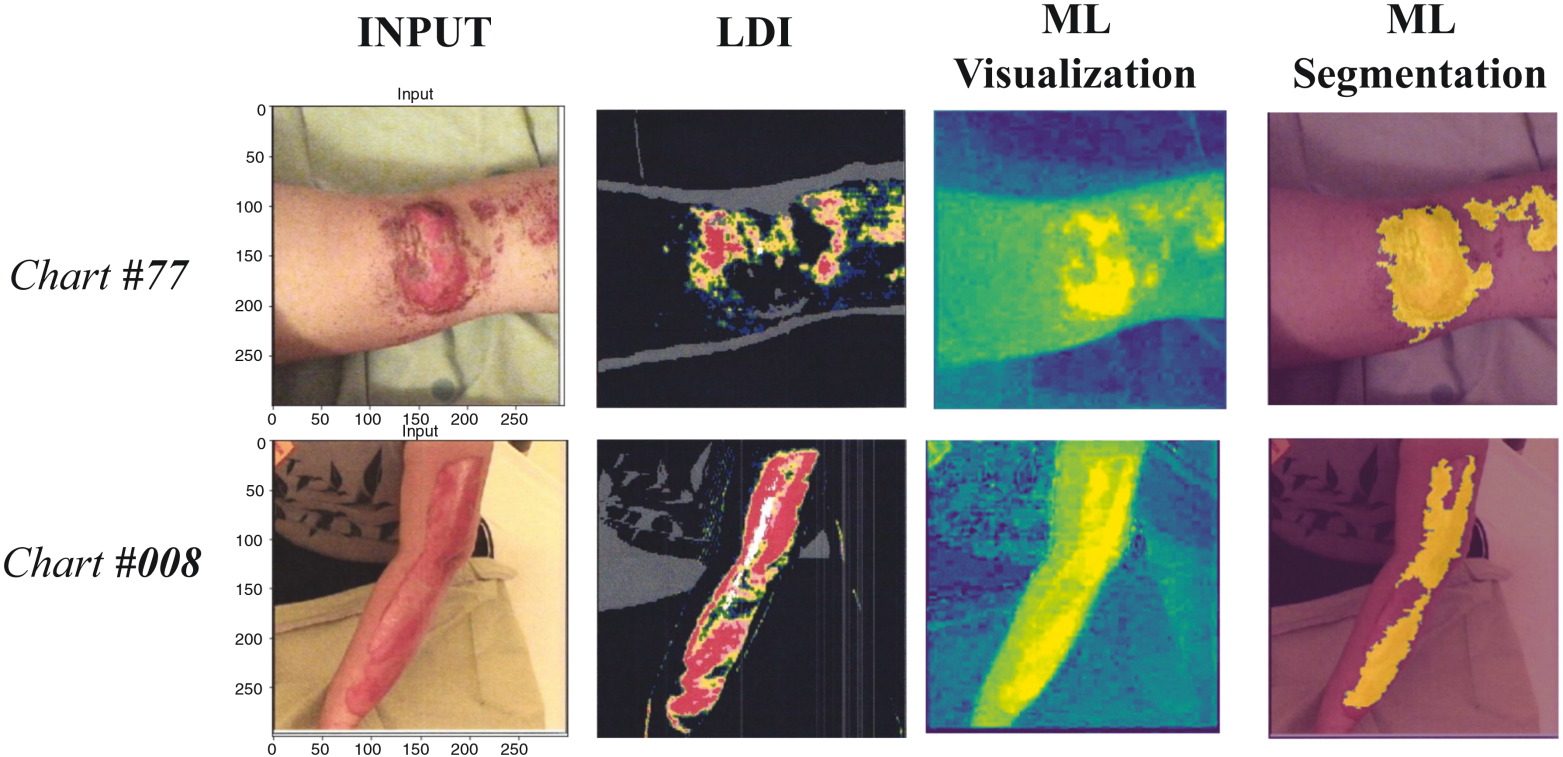
A Visual Representation of Boundary Attention Mapping Analysis and Statistical Analysis. A Clinical Photo (600 × 600 Pixels) Was Tested With our AI/ML Burn Assessment Tool. The Total Surface Area of the Burn Our AI/ML Tool Recognized (ML Visualization) Was Represented Through a Mask Image Composed of Pixels. A Pixel-Wise Comparison Was Completed With LDI Image by Directly Overlaying Both Images. The AI/ML Tool Was Able to Pick Up on 91.6% of Pixels That LDI Assessed to be a Burn Injury, Which Showed 78.17% Sensitivity and 93.7% Specificity

An LDI-paired dataset comprising a total of 184 2D skin burn images, accompanied by their corresponding LDI scans, from 104 burn patients in our province was selected for further LDI analysis based on clinical decisions. This second dataset, created by further preprocessing of images and scans into an aligned binary dataset to allow for pixel-wise comparison between AI and LDI was used to calculate the accuracy, specificity, and sensitivity of the BAM system, when ascertaining a patient’s burn area. When comparing the outcomes of our computer-vision-based saliency mapping method, referred to as BAM, with those of LDI in terms of injury boundary measurement, our method’s burn area segmentations attained an accuracy of 91.60%, sensitivity of 78.17%, and specificity of 93.37% (Figure 3).

**Figure 4.**
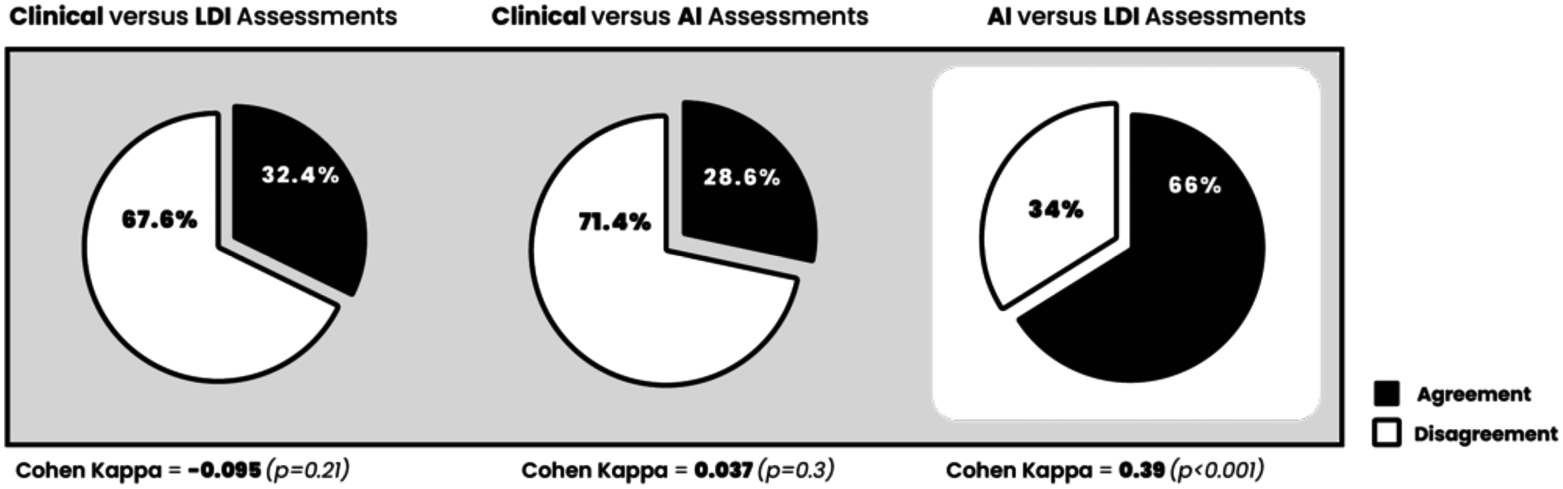
Agreement Analysis Between Clinical, LDI, and AI Burn Assessment. Cohen’s Kappa Analysis (Linear Weights) Was Completed to Assess Inter-Rater Reliability Between 3 Assessment Methods (Table 3). Clinical Vs LDI Assessment Had the Same Depth Assessment on 32.4% of Burn Images Tested With a Kappa Value of 0.037 (*P* = .3), Which Indicated Poor Agreement (A). Clinical Vs AI Assessment Had the Same Depth Assessment on 28.6% of Burn Images Tested With a Kappa Value of −0.095 (*P* = .21), Which Also Showed Poor Agreement (B). However, AI Vs LDI Assessment Had the Same Depth Assessment on 66% of Burn Images Tested With a Kappa Value of 0.39 (*P* < .001), Which Represents Fair Agreement (C)

To analyze the accuracy of clinical, LDI, and AI burn severity assessments, a slightly larger dataset of 138 patients with 241 LDI scans was used. In the above exploratory data analysis section, we reported burn assessment that underwent clinician and LDI assessment. An agreement analysis was performed, where clinical vs LDI analysis agreed 29% of the time and clinical vs AI analysis agreed 28.6% of the time, while the agreement between AI vs LDI burn severity assessments was 67% (Figure 4). Cohen’s kappa analysis of the 3 comparisons showed 0.037 (*P* = .30), −0.095 (*P* = .21), and 0.37 (*P* < .001) respectively, showing poor agreement between clinical vs LDI and AI, but fair agreement between AI and LDI assessments.

## DISCUSSION

Herein, this experimental study demonstrated the clinical utility of our previously published AI tool (or CNN-BAM) in thermal burn assessments. The CNN was trained using 1684 skin burn images. The accuracy of the CNN-BAM tool was measured in 2 domains: (1) accuracy of burn depth assessment and (2) accuracy of burn area assessment by comparing CNN-BAM outcomes against 184 clinical and its corresponding LDI photos. This was completed by reintroducing the clinical photo to our CNN-BAM tool, which showed comparable efficacy of burn depth and area assessment compared to the LDI scan. These results together provide an alternative burn assessment tool that can be applied in an accessible mobile application.

The field of AI/ML in medicine, particularly in burn assessment, has witnessed a significant surge in research and development over the past decade.^13^ Of various sophisticated models in AI/ML being applied, CNNs are largely used for image-based tasks, which is particularly relevant in burn assessment.^14^ These CNN models are made up of blocks of convolutional layers that are adept at extracting image features (eg, colors, edges, and texture). CNNs “learn” about a class of objects (eg, what skin with superficial depth burns looks like) from training images, which are labeled with the attribute that one wants to “teach” the model. These image labels are usually annotated by experts in the field, for example, burn surgeons and providers for burn images. In other words, CNNs primarily follow the paradigm of “supervised learning” where humans, by way of labeled images, guide how the ML model learns. These CNN models use differing neural architectures that are optimized to extract desired features from images to generalize and appropriately label images it has not previously “seen.”

There are various studies that have successfully developed a deep CNN using a dataset of burn images and shown accuracy in burn assessment up to 96%.^12,17,18^ A critique of these studies is the difficulty in comparing the accuracy of these AI/ML tools against other assessment aids. Thatcher et al. described a significant divergence from the above studies, by their application of multispectral images as the learning dataset, instead of high-resolution polarized or light photography.^19^ In their study, data was collected from 38 subjects, with a total of 58 burns. For each burn site, 2 critical procedures were followed: a clinical diagnosis to ascertain the burn depth, and a tissue biopsy to validate this diagnosis. This information was then applied to various CNN architectures, including Unet, SegNet, and dFCN, as well as a voting ensemble approach using these architectures. The performance of these algorithms was rigorously evaluated using Leave-One-Out Cross-Validation, a method that ensures thorough testing by training the model on all data points except one that is used for testing. The most effective algorithm in their study was the ensemble, which demonstrated an impressive 81% sensitivity and an exceptional 100% specificity, coupled with a 97% positive predictive value. This high level of accuracy showcases the potential of multispectral imaging in conjunction with advanced neural architectures in the accurate assessment of burn injuries.

Our approach to AI-assisted burn assessment differs from the previously described methods in the neural architecture, training, and testing phases of our model. The machine learning approach used EfficientNet-B7 architecture of CNNs. Compared to other CNNs, EfficientNet has been reported to perform exceptionally well with skin images, even when the dataset is small to medium sized, while processing mobile-captured images with potential noise (such as images with background in the field and low-resolution images).^20,21^ Utilization of EfficientNet makes this tool appropriate to be embedded in an application form that can interpret images captured from mobile phones. The goal of our mobile application is to be used at multiple stages of the burn injury healthcare continuum. First, this tool can be used by first responders, when initially assessing a patient with a thermal injury post-de-epithelialization. The mobile application will guide the user through initial burn resuscitation, like that of Advanced Burn Life Support. Thereafter, clinical contextual information will be obtained, including demographic data, events leading up to injury, and mechanism of injury. Ultimately, clinical photo(s) will be obtained of the injured region. The embedded AI tool will compute the depth of injury as well as TBSA and suggest whether the patient can be seen by primary care at the next earliest outpatient appointment, must go to a local acute treatment center, or should transferred to the nearest burn center. In addition, this tool also has the potential to be used in rural, remote, and low-resource communities globally, where adjunct thermal injury assessment such as LDI and other technologies may not be available due to resource constraints. Accurate burn assessments in these settings would aid appropriate triaging to determine whether the patient would benefit from transfer and treatment at a specialized burn center in a timely manner. Ultimately, it is the goal that this tool can be available in areas that solely rely on clinical assessments, as an efficient, cost-effective alternative to previously described assessment aids.

Like previous studies,^12,17,18^ our model was also exclusively trained on images annotated with clinical assessments. However, this model differs in that we validated our tool by comparing its accuracy against another adjunct burn assessment tool that has shown high accuracy and utility at various burn centers across the world: LDI. The comparative results of the AI tool described, in addition to the use of a novel saliency system that segments burn areas, allow our system to (1) provide accurate burn assessment and (2) provide precise mapping of burn regions. These results not only underscore the accuracy of our AI model but also set a strong foundation for its clinical validation as a triage tool for LDI assessments in the future.

Future directions include clinical validation of this tool in a large multi-center trial. Our AI tool has been embedded into a software and a prototype mobile application has been developed. The plan is to launch this product and validate its utility at the Acute Burn Clinic, a regional catchment clinic for new patients with burns are referred by self-referral, emergency departments, and primary care physicians. To further validate this, a prospective experimental study can be conducted on new patients with burns who undergo AI assessment can be pathologically confirmed using a biopsy. A multi-centered approach would capture a more diverse patient demographic. Furthermore, the application of the EfficientNetB7 neural network to other components of burn care and skin conditions, including assessment of scars, wound healing progression, and/or malignant skin lesions can also be explored.

One limitation of this study is the inconsistency of assessment outcomes from the 3 modalities: clinical, LDI, and AI. While clinical and AI assessments are reported in terms of depth or severity of the burn, LDI analysis results are reported in terms of healing potential (in days). Thus, the timeframes of healing potential are extrapolated for equivalence to burn depths. Moreover, the initial inclusion of burn images into the study was based on the interpretation of LDI color map scans. Also, despite a moderately sized benchmark dataset with 271 LDI scans from 138 patients, the majority of the population’s Fitzpatrick score was Type I–III. As the patient’s skin tones were not differentiated during the learning phase, this may impact the accuracy of our technology for populations with higher Fitzpatrick scores. This provides future opportunities to increase the sample size with greater diversity in the patient population in order to train our algorithm to assess thermal injuries no matter the skin tone. Lastly, AI is relatively novel in clinical settings, and its utility continues to be investigated. While this study demonstrates promising results for the application of AI, the authors suggest it does not replace evidence-based clinical judgment. As such, its implementation should be cautiously introduced as an adjunct to clinical assessment of thermal injuries with appropriate validation.

## CONCLUSION

This study describes a novel clinically validated adjunct burn assessment tool using a type of CNN that is tailored to be applied in a mobile application format. This tool has shown comparable accuracy in assessing burn depth and area of injury to that of LDI imaging. This tool represents an inexpensive, easy-to-use, versatile tool that can be used in all healthcare settings to help clinicians determine the course of patient management. Future studies will examine its utility in clinical settings and patient outcomes.

## References

[1] Markiewicz-Gospodarek A , Kozioł M, Tobiasz M, Baj J, Radzikowska-Büchner E, Przekora A. Burn wound healing: clinical complications, medical care, treatment, and dressing types: the current state of knowledge for clinical practice. Int J Environ Res Public Health. 2022;19(3):1338. 35162360 10.3390/ijerph19031338 PMC8834952

[2] Kwan P , Hori K, Ding J, Tredget EE. Scar and contracture: biological principles. Hand Clin. 2009;25(4):511–528. 19801124 10.1016/j.hcl.2009.06.007

[3] Abazari M , Ghaffari A, Rashidzadeh H, Badeleh SM, Maleki Y. A systematic review on classification, identification, and healing process of burn wound healing. Int J Low Extrem Wounds. 2022;21(1):18–30. 32524874 10.1177/1534734620924857

[4] Wong J , Lin W, Ding J, Tredget EE. Prevention and management of scarring after thermal injury. Plast Aesthet Res. 2021;8:9.

[5] Zuo KJ , Medina A, Tredget EE. Important developments in burn care. Plast Reconstr Surg. 2017;139(1):120e–138e. 10.1097/PRS.0000000000002908 28027250

[6] Claes KEY , Hoeksema H, Robbens C, et al The LDI enigma, part I: so much proof, so little use. Burns. 2021;47(8):1783–1792. 33658147 10.1016/j.burns.2021.01.014

[7] Jaskille AD , Shupp JW, Jordan MH, Jeng JC. Critical review of burn depth assessment techniques: part I. Historical review. J Burn Care Res. 2009;30(6):937–947. https://doi.org/10.1097/BCR.0b013e3181c07f21 19898102

[8] Jaskille AD , Ramella-Roman JC, Shupp JW, Jordan MH, Jeng JC. Critical review of burn depth assessment techniques: part II. Review of laser Doppler technology. J Burn Care Res. 2010;31(1):151–157. Preprint at https://doi.org/10.1097/BCR.0b013e3181c7ed60 20061851

[9] Hop MJ , Stekelenburg CM, Hiddingh J, et al; LDI Study Group. Cost-effectiveness of laser Doppler imaging in burn care in the Netherlands: a randomized controlled trial. Plast Reconstr Surg. 2016;137(1):166e–176e. 10.1097/PRS.0000000000001900 26710049

[10] Ethier O , Chan HO, Abdolahnejad M, et al Using computer vision and artificial intelligence to track the healing of severe burns. J Burn Care Res. 2022;45(3):700–708. https://doi.org/10.1093/jbcr/irad197 38126807

[11] Abdolahnejad M , Lee JJ, Chan HO, et al Boundary Attention Mapping (BAM): fine-grained saliency maps for segmentation of Burn Injuries. arXiv. 2023. eprint 2305.15365.

[12] Suha SA , Sanam TF. A deep convolutional neural network-based approach for detecting burn severity from skin burn images. Machine Learning with Applications. 2022;9(6):100371.

[13] Moura FSE , Amin K, Ekwobi C. Artificial intelligence in the management and treatment of burns: a systematic review. Burns and Trauma. 2021:9:tkab022. 10.1093/burnst/tkab022. eCollection 2021. 34423054 PMC8375569

[14] Ferdinand J , Viriya Chow D, Yuda Prasetyo S. Automated skin burn detection and severity classification using YOLO Convolutional Neural Network Pretrained Model. In: E3S Web of Conferences. Vol. 426. EDP Sciences; Jakarta, Indonesia. 2023.

[15] Robb L. Potential for machine learning in burn care. J Burn Care Res. 2022;43(3):632–639. 34643694 10.1093/jbcr/irab189

[16] Taib BG , Karwath A, Wensley K, Minku L, Gkoutos GV, Moiemen N. Artificial intelligence in the management and treatment of burns: a systematic review and meta-analyses. J Plast Reconstr Aesthet Surg. 2023;77(Feb):133–161. 36571960 10.1016/j.bjps.2022.11.049

[17] Cirillo MD , Mirdell R, Sjöberg F, Pham TD. Improving burn depth assessment for pediatric scalds by AI based on semantic segmentation of polarized light photography images. Burns. 2021;47(7):1586–1593. 33947595 10.1016/j.burns.2021.01.011

[18] Cirillo MD , Mirdell R, Sjöberg F, Pham TD. Time-independent prediction of burn depth using deep convolutional neural networks. J Burn Care Res. 2019;40(6):857–863. 31187119 10.1093/jbcr/irz103

[19] Thatcher JE , Squiers JJ, Kanick SC, et al Imaging techniques for clinical burn assessment with a focus on multispectral imaging. Advances in Wound Care. 2016;5(8):360–378. https://doi.org/10.1089/wound.2015.0684 27602255 PMC4991589

[20] Venugopal V , Raj NI, Nath MK, Stephen N. A deep neural network using modified EfficientNet for skin cancer detection in dermoscopic images. Decis Anal J. 2023;8(Sept):100278.

[21] Mhedbi R , Credico P, Chan HO, Joshi R, Wong JN, Hong C. A Convolutional Neural Network based system for classifying malignant and benign skin lesions using mobile-device images. medRxiv. 2023. 10.1101/2023.12.06.23299413

